# Recent Progress of Droplet Microfluidic Emulsification Based Synthesis of Functional Microparticles

**DOI:** 10.1002/gch2.202300063

**Published:** 2023-08-11

**Authors:** Fei Long, Yanhong Guo, Zhiyu Zhang, Jing Wang, Yong Ren, Yuchuan Cheng, Gaojie Xu

**Affiliations:** ^1^ Department of Mechanical Materials and Manufacturing Engineering University of Nottingham Ningbo China Ningbo 315100 P. R. China; ^2^ Center of Materials Science and Optoelectronics Engineering University of Chinese Academy of Sciences Beijing 100049 P. R. China; ^3^ Zhejiang Key Laboratory of Additive Manufacturing Materials Ningbo Institute of Materials Technology and Engineering Chinese Academy of Sciences Ningbo 315201 P. R. China; ^4^ Research Group for Fluids and Thermal Engineering University of Nottingham Ningbo China Ningbo 315100 P. R. China; ^5^ Nottingham Ningbo China Beacons of Excellence Research and Innovation Institute Ningbo 315040 P. R. China; ^6^ Department of Electrical and Electronic Engineering University of Nottingham Ningbo China Ningbo 315100 P. R. China; ^7^ Key Laboratory of Carbonaceous Wastes Processing and Process Intensification Research of Zhejiang Province University of Nottingham Ningbo China Ningbo 315100 P. R. China

**Keywords:** droplet microfluidics, emulsions, functional microparticles, microcapsules, multiphase system

## Abstract

The remarkable control function over the functional material formation process enabled by droplet microfluidic emulsification approaches can lead to the efficient and one‐step encapsulation of active substances in microparticles, with the microparticle characteristics well regulated. In comparison to the conventional fabrication methods, droplet microfluidic technology can not only construct microparticles with various shapes, but also provide excellent templates, which enrich and expand the application fields of microparticles. For instance, intersection with disciplines in pharmacy, life sciences, and others, modifying the structure of microspheres and appending functional materials can be completed in the preparation of microparticles. The as‐prepared polymer particles have great potential in a wide range of applications for chemical analysis, heavy metal adsorption, and detection. This review systematically introduces the devices and basic principles of particle preparation using droplet microfluidic technology and discusses the research of functional microparticle formation with high monodispersity, involving a plethora of types including spherical, nonspherical, and Janus type, as well as core–shell, hole–shell, and controllable multicompartment particles. Moreover, this review paper also exhibits a critical analysis of the current status and existing challenges, and outlook of the future development in the emerging fields has been discussed.

## Graphical abstract

Overview of latest advances in droplet microfluidic formation of highly monodispersed microparticles, involving a plethora of types including spherical, nonspherical, and Janus type, as well as core–shell, hole–shell, and controllable multicompartment particles, is provided. The critical analysis of the current status and existing challenges is also conducted, and the future development in the emerging fields by microfluidic emulsification is envisioned.

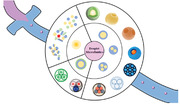

## 1 Introduction

Microparticles collectively refer to entities of different shapes on the micrometer scale and nanometer scale.^[^ ^1^ ^]^ In order to meet the hot demand in the fields of chemical engineering,^[^ ^2^, ^3^ ^]^ materials,^[^ ^4^ ^]^ medicine and biology,^[^ ^5^ ^]^ microparticles are endowed with functionality and named as functional microparticles. Generally, the overall function of microparticles largely depends on their structure and constituent materials. To ensure that each microparticle has uniform and controllable properties, it is crucial that the uniformity of size and shape determines the physicochemical properties of microparticles,^[^ ^6^ ^]^ especially packing characteristics, encapsulation amount and its releasing kinetics.^[^ ^7^ ^]^ According to different requirements, a variety of microspheres with different structures and functions can be derived. Microparticles with a porous structure can provide a porous confinement space for drug or phase change material embedding,^[^ ^8^ ^]^ cell adhesion,^[^ ^9^ ^]^ and proliferation.^[^ ^10^ ^]^ Due to large specific surface area, it can also be applied for the adsorption of substances, so it has a wide range of applications in drug delivery, phase change energy storage, adsorption separation, and other fields.^[^ ^11^, ^12^ ^]^ On the other hand, microparticles with a cavity structure can provide a closed cavity space for the encapsulation and protection of active substances (e.g., drugs, enzymes, etc.), and the controllable release of encapsulated substance is achieved through the change of the physical and chemical properties of the functional components in the capsule wall.^[^ ^13^ ^]^ Therefore, it plays an important role in the fields of substance encapsulation, controlled release, and immobilized enzyme reaction.

**Figure 1:**
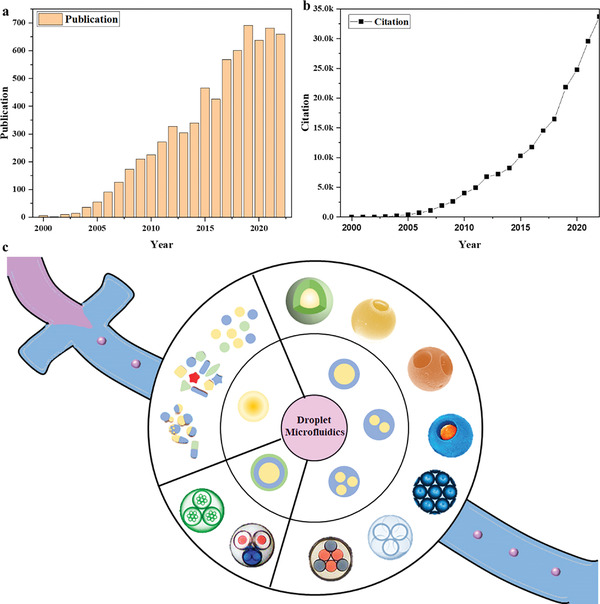
The statistics data on the topic of droplet microfluidics from Web of Science for a ) publication b) citation. c) The overview of droplet microfluidics.

Traditional microparticle preparation techniques such as precipitation polymerization,^[^ ^14^ ^]^ spray drying,^[^ ^15^ ^]^ layer‐by‐layer self‐assembly,^[^ ^16^ ^]^ etc., are usually difficult to realize the controllable construction of functional microparticles with uniform and controllable size, as well as diverse structures, compositions, and shapes. Microfluidic technology, which has developed rapidly since the 1990s, is a technology for manipulating a small amount of volumes in microscale channels.^[^ ^17^ ^]^ No matter how microfluidic technology changes, as the name suggests, it is always a combination of “microfluidic” and “control”; and, the former belongs to flow channel design, while the latter belongs to instrument. The combination of these two completes the precise control and operation of single‐phase or multiphase fluids. In terms of droplet microfluidic technology, immiscible liquids are utilized to form monodisperse microspheres with controllable particle size and uniform distribution under the combined action of pressure, fluid shear force, and surface tension.^[^ ^18^ ^]^ In recent years, droplet microfluidic technology has been significantly improved, mainly reflected in the following aspects. From Web of Science, the number of publications and citations of related articles on the topic of droplet microfluidics has increased year by year, as shown in **Figure** **1**a,b. Benefiting from the continuous introduction of novel materials and fabrication techniques, the device assembly of droplet microfluidics has undergone a transitional development from simple 2D microchannels^[^ ^19^ ^]^ to complex 3D systems^[^ ^20^, ^21^ ^]^ that is easier carry out hydrophilic or hydrophobic treatment on the wall surface. With respect to droplet dynamics, the generation of microfluidic droplet has been intensively explored, including passive hydrodynamic pressure^[^ ^22^ ^]^ or active externally driven generation.^[^ ^23^ ^]^ The fluid dynamics contribute to the accurate control of droplets and multiphase interfaces, which enable the system designs for generating and manipulating droplets with diverse behaviors and morphologies.^[^ ^24^ ^]^ By virtue of these theoretical and technological advances, droplet microfluidics has broad value in the application of biochemical analysis^[^ ^25^ ^]^ and generation of nano‐ and microscale materials.^[^ ^26^, ^27^ ^]^ Therefore, microfluidic technology has shown incomparable advantages over other technologies in the continuous and controllable construction of monodisperse functional microparticles with various structures and functions.

Till present, in the aspect of droplet microfluidics, the development of biochemical analysis^[^ ^28^ ^]^ and detection technology^[^ ^29^, ^30^ ^]^ have been summarized vis reviewing the basic principles^[^ ^31^ ^]^ and fundamental applications.^[^ ^32^ ^]^ Differently, this review focuses on recent developments in the preparation of novel functional microparticles by droplet microfluidics and their emerging applications, as shown in Figure 1c. First, the conventional methods for manufacturing functional microparticles were compared and studied, which highlight the advantages of droplet microfluidics. Then, the devices and fabrication mode of droplet microfluidics were introduced to provide more sufficient reference for future research. Finally, the research status of microfluidic method for preparing microparticles with different structures and functions through single or double emulsion was emphatically expounded, which provides novel ways and guidance for the further design functional microparticles.

## 2 The Conventional Approaches for Fabricating Microparticles

In the last decades, with the continuous expansion scale of the additive manufacturing industry, the demand for functional microparticles has been increasing. In detail, the preparation methods can be divided into three types that of physical, chemical, and physical–chemical ways. All the mentioned approaches have been compared and analyzed in **Table** **1**.

**Table 1:** The comparison and analysis of conventional fabrication approaches

Methods	Droplet type	Advantages	Disadvantages	Refs.
Emulsion solvent evaporation	Single, double	Mild reaction conditions Easy to control	Recover organic solvents Time consuming Low production intensity	[28, 29]
Phase separation	Single	Cost effective Batch processing	Difficult to disperse microparticles Lack of sterility	[32]
Spray drying	Single	Fast production rate Simple Process Large‐scale production	High energy consumption Hard to control thermal stress	[15, 53]
Electrostatic spraying	Double	Easy to operate High coating forming rate	Poor repeatability	[54]
Solvent evaporation	Hollow, porous	Easy to operate Good repeatability	Toxic solvent	[55, 56]
Template	Hollow	Constructive Easy to operate	Poor stability Low efficiency	[57]
Suspension polymerization	Porous	Stable distribution Easy to handle Low cost	Uneven particle size Cumbersome process	[58, 59]

### 2.1 Single Solid Microparticles

#### 2.1.1 Emulsion Solvent Evaporation Method

**Figure 2:**
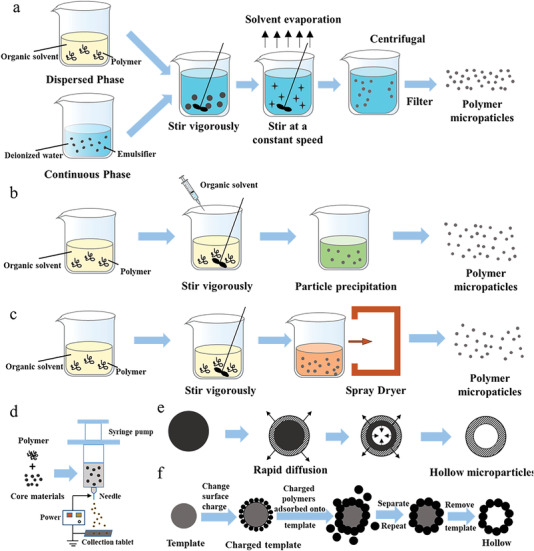
The procedure of generating microparticles by a) emulsion solvent evaporation, b) phase separation, c) spraying drying, d) electrostatic spraying, e) solvent evaporation, and f) template method.

The emulsion solvent evaporation method is one of the common physical methods for manufacturing microspheres, which is relative mature with simple operation.^[^ ^33^ ^]^ As its reaction conditions are mild, the process parameters are convenient to control.^[^ ^34^ ^]^ The preparation process can be simply summarized as dissolving and dispersing the drugs in purified water or organic solvent, then emulsifying with a polymer water phase, and finally injecting into the external water phase (**Figure** **2**a). With the diffusion and volatilization of the organic solvent, the cured microspheres can be obtained. Zhao et al.^[^ ^35^ ^]^ has fabricated isoperidone microspheres by this method with the carrier material of polylactic‐*co*‐glycolic acid (PLGA), and the obtained sooth‐surfaced microspheres had an average particle size of 82 µm and an encapsulation efficiency of 92%. However, the disadvantages mainly lie in the need for recovery of organic solvents, and it requires a long time for solvent evaporation, resulting in low production intensity.

#### 2.1.2 Phase Separation Method

The phase separation method is to dissolve, disperse or mix the drugs as the form of emulsion droplets in the organic solvent with polymer, and then a phase separation reagent is added into (Figure 2b). Due to the decrease of solubility, the polymer precipitates to form small droplets of the condensed phase.^[^ ^36^ ^]^ Finally, the extractant is introduced to remove the organic solvent and phase separation reagent, and then microparticles are obtained by solidification. The essence of this method is extraction technology, which belongs to physical–chemical phenomenon. The properties of microparticles are effect by a few of factors,^[^ ^37^ ^]^ such as the intermolecular interaction between polymer materials, the rate and duration of each step, etc. The method does not require expensive equipment that is easy to process in batches, while the generated microparticles aggregate together that are difficult to disperse. Moreover, because of high residue of organic solvent, the sterility is difficult to guarantee. With this method, Chen et al.^[^ ^38^ ^]^ have prepared triptorelin acetate microspheres whose encapsulation efficiency of 71.35% by optimizing various formulation factors.

#### 2.1.3 Spray Drying Method

Spray drying is a method that liquid auxiliary materials are sprayed into a hot drying medium for the conversion of dry powder.^[^ ^15^ ^]^ As shown in Figure 2c, the specific preparation process can be divided into three procedures. 1) The atomizer atomizes the solution of auxiliary materials into small droplets. 2) The drying gas solidifies and dries the small droplets. 3) The dry microparticles are separated from the spry dryer. In addition to fast solvent drying speed, this preparation is simple with fast production rate, which is suitable for large‐scale industrial production. However, the large volume of instrument leads to high energy consumption, and this process requires thermal stress that is hard to control. If the temperature is too high, the microparticles will deform and aggregate easily; while the temperature is too low, the solvent residue will be high, resulting in poor control of the particle size. Shi et al.^[^ ^39^ ^]^ created leuprolide PLGA microparticles by spray drying. When the polymer concentration is low, the morphology is spherical; if concentration is too high, irregular particles may appear.

### 2.2 Double‐Layer Microparticles

#### 2.2.1 Modified Emulsion Solvent Evaporation

Since the double‐layer microparticles require two types of polymers, the preparation method has been improved on the basis of the emulsion solvent evaporation method. Based on the different solubility of two polymers in solvents, a poor solvent for the core polymer is introduced into the mixed solutions, so that the core polymer is solidified first, and then the shell polymer is solidified and wrapped the core to form double‐layer microparticles.^[^ ^40^ ^]^ Taking a particular example, Naraharisetti et al.^[^ ^41^ ^]^ have constructed gentamicin double‐layer microspheres with the particle size between 100 and 600 µm, in which the shell material is PLGA (soluble in both dichloromethane and ethyl acetate) and the core material selected poly‐l‐lactic acid (PLLA, soluble in dichloromethane but not ethyl acetate). At first, PLLA and PLGA were dissolved in dichloromethane and ethyl acetate, respectively. Then, these two polymer solutions were ultrasonically mixed, and since PLLA was insoluble in ethyl acetate, small semisolid droplets would spontaneously form with PLGA surrounding them distribution. Finally, transferring to the external aqueous phase, with the volatilization and diffusion of solvent, PLLA at the core layer was precipitated first and PLGA at the outer layer was subsequently solidified.

#### 2.2.2 Electrostatic Spraying Method

Electrostatic spraying, also called electrohydrodynamic atomization,^[^ ^42^ ^]^ is composed of three parts that are an external electric field, a syringe pump and a collection tablet (Figure 2d). Under the external electric field, the polymer solution obtains electrostatic force; when it is greater than surface tension caused by droplets, the solution will be ejected from the tip of nozzle. Small droplets with the same charge are dispersed to the collection tablet under the action of Coulomb repulsion, and then microparticles are obtained after drying and solidifying. Recently, Zhou et al.^[^ ^43^ ^]^ have constructed double emulsion microparticles with polyvinyl alcohol (PVA) and polycaprolactone as inner core and outer shell, respectively, by electrostatic spraying for the entrapment of doxorubicin.

### 2.3 Hollow Microparticles

#### 2.3.1 Solvent Evaporation Method

As a common method for preparing hollow microparticles, its specific process resembles emulsion solvent evaporation method,^[^ ^44^ ^]^ and the formation mechanism has been shown in Figure 2e. Generally, two kinds of solvents are applied to mix and dissolve the carrier materials, and the common selected mixture is ethanol/dichloromethane. In the stage of solidification and drying, the solvent with higher solubility or better volatility will be diffused and removed quickly in the continuous phase, so that the polymer outside the emulsion droplet can form a thin film after precipitation, solidification, and deposition. Another solvent volatilizes and diffuses slowly with the solidification of internal polymer, shrinking from inside to outside for a cavity in center. Zhu et al.^[^ ^45^ ^]^ have prepared dipyridamole hollow microspheres with excellent floating properties by solvent evaporation using ether and ethanol as solvents, ethyl cellulose, and carbomer as carrier materials.

#### 2.3.2 Template Method

The template method is characterized in that the shell materials is adsorbed and solidified onto template driven by hydrogen bonds and electrostatic interactions.^[^ ^46^ ^]^ As shown in Figure 2f, the hollow microparticles can be obtained by removing template after solvent dissolution and high‐temperature calcination. Polyaniline hollow microspheres were prepared by Li et al.^[^ ^47^ ^]^ using poly‐2‐acrylamide‐2‐methylpropanesulfonic acid as a template, with an average particle size of ≈410 nm.

### 2.4 Porous Microparticles

#### 2.4.1 Modified Solvent Evaporation Method

The modified method is that the porogen are required for the preparation of porous microparticles, and the suitable porosity can be produced by adjusting the type and concentration of the porogen.^[^ ^48^ ^]^ There are various types of porogens, mainly gas‐generating porogens (e.g., ammonium bicarbonate,^[^ ^49^ ^]^ etc.), osmotic porogens (e.g., sodium chloride, potassium chloride, etc.), extractable porogens, and pore agents (Pluronic,^[^ ^50^ ^]^ etc.).

#### 2.4.2 Suspension Polymerization

The suspension polymerization method is to suspend polymerization monomer, initiator, and porogen in the liquid phase matrix for polymerization reaction and remove the porogen after the reaction is complete. Commonly used porogens in the reaction are organic solvents and linear polymers. Cai et al.^[^ ^51^ ^]^ prepared porous microspheres containing epoxy groups by suspension polymerization, and the porosity can be up to 62.45%. Although this method is currently the most common method for manufacturing porous microspheres, it requires artificial conditions such as mechanical stirring or oscillation or the action of a dispersant to disperse the liquid phase into droplets. The instability of the force during the stirring process results in uneven particle size of the microspheres. Residual porogen needs to be removed after pelleting, which requires multiple cleanings, and the process is very cumbersome.^[^ ^52^ ^]^

## 3 Droplet Microfluidics Devices

**Figure 3:**
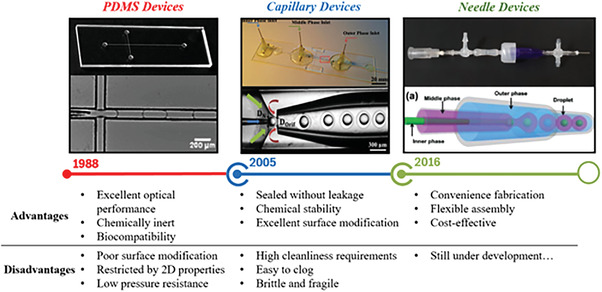
The development process of droplet microfluidics over time with their advantages and disadvantages.

Microfluidics can be regarded as a technique for systematically manipulating and controlling fluids in channels on the scale of several micrometers to hundreds of micrometers. In the 1950s, Skegges and Hochstrasser^[^ ^60^ ^]^ have proposed a compartmentalized continuous flow technique for analytical chemistry experiments in fluid pipelines. There are three significant branches in the field of microfluidics technology: droplet, digital, and continuous microfluidics. Among them, droplet microfluidics is an important way for fabricating the functional particles that can be synthesized by taking the emulsion droplet system with the closed liquid–liquid phase interface. Droplet microfluidics technology was first proposed by Thorsen et al.,^[^ ^61^ ^]^ and then the related research results about microparticles with excellent biocompatibility have been reported by Nie et al.^[^ ^62^ ^]^ in the medical field. Subsequently, a series of droplet microfluidic devices have been constructed with various materials, for example, glass, quartz, plastics, crystalline silicon, and polydimethylsiloxane (PDMS). To observe the process and shape of droplet formation, the required materials need transparent. The development processes of droplet microfluidics over time with their advantages and disadvantages are summarized in **Figure** **3**.

### 3.1 PDMS Based

The most commonly used method for manufacturing PDMS‐based microfluidic chips is soft photolithography^[^ ^63^ ^]^ that refers to using a patterned PDMS^[^ ^64^ ^]^ as a stamp or replica mold to transfer features onto the desired substrate. The whole process has been schematized step by step, which can be in a mass production. Owing to the low surface energy of PDMS, it is convenience to remove from the mold after curing without the damage to the mold. With the emergence of customization, the manufacturing method begins transferring to additive manufacturing gradually.^[^ ^65^ ^]^ As PDMS has excellent optical properties and it can transmit ultraviolet light with a wavelength above 300 nm and visible light in all bands, it is suitable materials for observing the real‐time status of microparticles generation. Owing to the excellent chemical inertness and biocompatibility, it is safe and nontoxic without reaction with chemical reagents. In 1998, the first PDMS chip has been fabricated by Whitesides and co‐workers,^[^ ^66^ ^]^ in which the dispersed phase (DP) and continuous phase (CP) are sheared in the same microchannel. Nevertheless, when the fluid flows through the inner wall of microchannel, the hydrophobic molecules will be absorbed, which influence on the quantitative analysis during the experiment. Although the modified surface technology was used, it is difficult to achieve the desired effect. The elastoplasticity of PDMS determines that its microstructure is not as stable as that of other rigid materials, and it will swell or deform under the influence of organic solvents.^[^ ^67^ ^]^ Furthermore, their applications are limited by their 2D properties and high‐pressure resistance.

### 3.2 Glass Microcapillary Based

The glass capillary based microfluidic chips have been introduced in the early of 21st century,^[^ ^68^ ^]^ featuring true 3D geometry and excellent solvent resistance.^[^ ^67^ ^]^ Different from the PDMS microchips, this setup is composed by a 3D channel form, in which structural configuration is assembled by multiple glass microcapillaries. The walls of each component can be processed in surface treatment for modifying the surface wettability precisely. The whole process is sample and convenient, particularly, the sealing performance without leakage is guaranteed. Due to the stability of glass, there is no need to consider the reagent corrosiveness and substrate swelling during the fabrication procedure.^[^ ^69^ ^]^ In 2005, the double emulsion droplets were first fabricated using glass microcapillary chips by Weitz research group^[^ ^70^ ^]^ at Harvard University, thus ushering in the rapid development of glass microcapillary LOCs technology. The dispersed phase requires a separate microchannel to connect with the continuous flow pipeline, and then the flowing fluid is sheared into droplets at the interface. The preparation process has high efficiency and low consumption, and using this device, not only single‐core particles but also multicore emulsions can be prepared. By further increasing the number of nested stages, functional particles with more complex structures can also be produced.^[^ ^71^ ^]^ However, it requires extremely high dimensional accuracy and cleanliness of manufacturing instruments, and the glass microchannels are easily blocked during the experimental operation. Considering the fragile of glass capillaries, the manual fabrication with microforge and pipette puller restricts production quantity at a time during assembly.

### 3.3 Needle‐Based

In 2016, a practical and facile method for assembling microfluidic setup with off‐the‐shelf dispensing needles in different sizes, micro‐cross‐links, and T‐junctions was first presented in the report of Li et al.^[^ ^72^ ^]^ Since the needle is the most significant component, it will henceforth be simplified as needle‐based microfluidic chips. The reversible combination of different components also allows flexible design of multifunctional devices to generate single/dual/multiphase dispersed emulsions with high controllability. To improve the droplet productivity of this method, inspired by the parallel connection of PDMS devices, Lian et al.^[^ ^73^ ^]^ developed and modified the application of new functional components to connect single‐needle‐based microfluidic devices in parallel to expand the yield to fabricate functional droplets. In fact, this strategy will not result in any complexity to the scaled‐up system. The off‐the‐shelf microfluidic device is a cost‐effective, portable, recyclable, and versatile powerful microplate form for scientific and industrial applications. However, so far, there was a limited number of studies and applications of microcapsules fabricated by this method, such as CO_2_ capture microcapsules^[^ ^74^ ^]^ and water treatment porous microparticles.^[^ ^75^ ^]^

## 4 The Microchannel Geometries for Generating Microparticles

Altering the state of the continuously flowing liquid to promote the microparticles, in which the key to manipulation is controlling the surface energy of microfluidics. This preparation process belongs to the passive way, which means that only the hydrodynamic pressure exists without the external energy input.^[^ ^76^ ^]^ Through the structural design of microchannel of microfluidic chip, immiscible two‐phase or multiphase fluids are injected from the outside to realize the generation of droplets. Taking an example, if selecting water phase and oil phase as the continuous phase and dispersed phase, respectively, or reversed condition, the resulting emulsions are considered W/O or O/W. According to various geometric structures, it can be divided into two categories that are coaxial (typical forms are flow focusing and coflowing) and noncoaxial (typical one is T‐junction). The geometries structure of coflowing microfluidic devices is mainly assembled by glass capillaries, while T‐junction and cross flowing are fabricated by microfabrication technology, such as additive manufacturing or soft lithography on PDMS, polymethylmethacrylate, glass slides, etc.

### 4.1 T‐Junction

**Figure 4:**
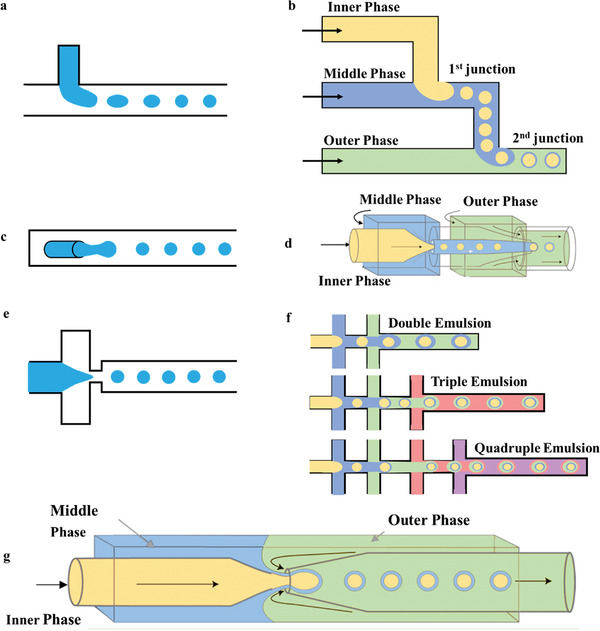
The structure of a) T‐junction, c) flow focusing, and e) coflowing. b) The double T‐junction, d) double flow focusing assemble, and f) multiple groups of coflowing units for multiple emulsion. G) The combination of coaxial flow structure and flow focusing type.

The principle of the T‐junction microfluidic system is that the primary and side flow are respectively applied to inject immiscible continuous phase and dispersed phase, which interest each other at an angle to form a cross flow. The inner phase is dispersed and broken into droplets by extrusion and shear of the outer phase (as shown in **Figure** **4**a). Thorsen et al.^[^ ^61^ ^]^ have reported the study on T‐junction microfluidic geometry first, in detail that the oil phase was fed into water phase with a laminar flow, forming monodisperse water droplets. Then, two serial T‐junctions made by Pyrex glass chips were applied to generate double emulsions, forming W/O droplets at the first hydrophobic junction followed by W/O/W droplets at the second hydrophilic junction (Figure 4b). In addition to ease of operation, T‐junction microfluidics is widely known for its sustainability, reproducibility, controllability, and affordability. However, due to the limitation of processing technology, the channel configuration of T‐junction is relatively common in 2D space. During the preparation of spherical microparticles, the T‐junction microfluidic system cannot maintain the fluids flowing through the uniform width and height, after considering the density and viscosity of flowing fluids. In typical, the length of resulting droplets is tenfold of the microchannel width. So, this fabrication mechanism restricts the generation of perfect droplets and related applications. To meet more droplet production requirements, V‐junction,^[^ ^77^ ^]^ and K‐junction^[^ ^78^ ^]^ have been introduced to modify T‐junction type.

### 4.2 Coflowing

The first application of coflowing microfluidic structures was at the macro level,^[^ ^79^ ^]^ and Cramer et al. have proposed to shrink down to the micron level to assemble capillaries to prepare droplets. In the process of device fabrication, the capillary coflowing method does not require photolithography technology or ultraclean laboratory for microchannel processing, which is relatively simple. Unlike the T‐shaped channel, the inertial effect cannot be ignored in coflow because it promotes the formation of droplets.^[^ ^80^ ^]^ A capillary is embedded in the microchannel for both in coaxial state; and the dispersed and continuous phase are introduced into capillary and microchannel, respectively. The liquid in dispersed phase can be squeezed and broken by the continuous phase at the outlet of the glass capillary with a ring shape, thus producing droplets (Figure 4c). Double emulsions are generated with two coflowing components^[^ ^81^ ^]^ in two emulsification steps. As shown in Figure 4d, the transition tube (thick‐walled circular capillary) connects the injection tube (circular capillary with tapered end) and the collection tube in a coaxial alignment. The destabilization of the droplets during preparation is called the Plateau–Rayleigh phenomenon that is tendency in interfacial area minimization according to interfacial area.^[^ ^82^ ^]^ It can be concluded that the Ca and flow rate are the primary factors, and two different droplet formation mechanisms are derived, which are the dripping principle and the jetting principle.^[^ ^83^ ^]^

### 4.3 Flow‐Focusing

The flow‐focusing configuration was first investigated by Anna et al.^[^ ^84^ ^]^ They prepared W/O monodisperse emulsions. On the basis of T‐junction microdevices, the two‐phase channel was improved to the cross‐shaped. The major difference is that the continuous phase will squeeze both sides of the dispersed phase at the intersection, and the dispersing flow will be sheared and broken at the “neck”, thereby generating droplets. In the case of keeping the liquid properties and the unchanged parameters of device structure, different experimental phenomena can be observed by changing flow rates. This type of device can not only fabricate droplets that have the same size with microchannel scale in the droplet mode, but also produce the tiny ones that is smaller than device scale in the jet mode (as shown in Figure 4e). Compared with the above two configurations, flow‐focusing is more suitable for generating multiphase droplets. Specially, two groups of immiscible liquids are focused on the orifice of the microchannel in the same or in the opposite direction. Under the action of shear force and interfacial tension, complex phase droplets can be formed, such as double emulsions generated by glass capillary microfluidic devices. A linear array of flow‐focused droplet makers is applied to generate droplets with multiple concentric liquid shells around a core drop, for instance, three units are used to produce a triple emulsion, and so on (Figure 4f).

As the demand for functional microparticles increasing, the microfluidic chip for preparing double emulsions can be formed by combining simple configurations. Combining the glass capillary chips with focusing type and coaxial flow structure can prepare double and complex emulsion (Figure 4g).^[^ ^70^, ^85^ ^]^ Here, Capillary number^[^ ^86^ ^]^ ($\mathrm{Ca}=\frac{\mu V}{\sigma }$, where *µ* is dynamic viscosity of working liquid, *V* is flowing velocity, and *σ* is the interfacial tension between two fluid phases) is brought in the state transition analysis. It can be concluded in three cases. 1) For Ca < 10^−2^, the shear force of dispersed phase cannot overcome the flow, and continuous phase is confined between the dispersed phase and the channel wall. With the increase of pressure, the dispersed phase enables to be squeezed and thus broken up by continuous phase into droplets. 2) For 10^−2^ < Ca < 10^−1^, the shear force exerted by continuous phase breaks the dispersed phase into droplets, and the droplet size can be controlled by adjusting Ca and the flow ratio between disperse and continuous phase. 3) For the case that Ca exceeds 10^−1^ (Ca > 10^−1^), jetting or coflowing regime is dominate, at which flow rate of dispersed phase is pretty larger than that of the continuous phase. It seems that T‐junction structure is difficult to process, and affected by shear force and surface tension, the generated droplets have poor stability with narrow range of droplet size. In addition, there are three modes of microdroplets generated in microchannels, which are dripping, jetting and stable coflow modes (**Table** **2**). The dipping mode enables to produce microdroplets in uniform size stably but with slow rate and low yield. So, jetting mode is usually used in the process of preparing functional particles.

**Table 2:** The regimes generated from three representative microfluidic structures

→Continuous phase →Dispersed phase	Dripping regime	Jetting regime	Stable coflow regime
T‐junction	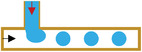	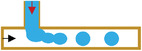	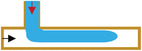
Coflowing	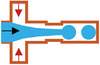	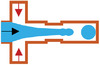	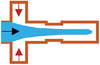
Flow focusing	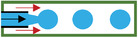	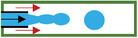	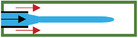

## 5 Monodisperse Functional Particles by Microfluidics

With the monodisperse emulsion droplets as a synthesis template, monodisperse microparticle functional materials with uniform size can be prepared, including single‐emulsion and multiemulsion microparticles. The precise control of microparticle size can be realized in a microscale range by changing the adjustable parameters with strong generality. In general, droplet uniformity is quantified with a coefficient of variation (CV),^[^ ^87^ ^]^ which describes the standard deviation from the mean droplet size

(1) $$\begin{array}{c} \mathrm{CV}\;\left(\%\right)=\frac{\sigma }{\bar{L_{d}}} \end{array}$$

where *σ* is the standard deviation of droplet length, and *L*_d_ is the mean of droplet length.

### 5.1 Single Emulsion Microparticles

**Figure 5:**
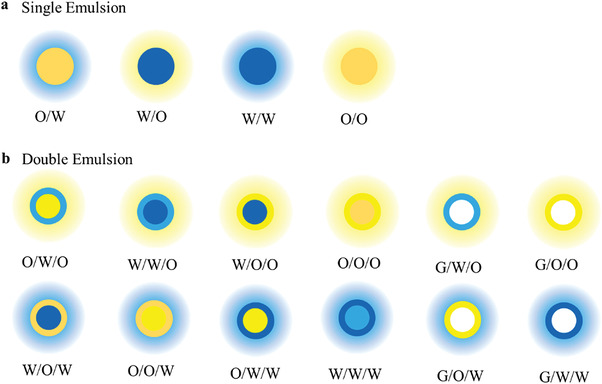
The groups of a) single emulsion and b) double emulsion.

The single emulsions are referring as the droplets that are the one phase dispersed in another immiscible phase. On the microscale, it can be divided into four groups (as shown in **Figure** **5**a). 1) Oil‐in‐water (O/W) is that oily droplets dispersed in a water base.^[^ ^88^ ^]^ 2) Water‐in‐oil (W/O)^[^ ^89^ ^]^ is that aqueous droplets dispersed in oily solution. 3) Water‐in‐water (W/W)^[^ ^90^ ^]^ is that water droplets are dispersed in an immiscible aqueous solution. 4) Oil‐in‐oil (O/O)^[^ ^91^ ^]^ is that oil droplets dispersed in an immiscible oily solution. After curing, single emulsion droplets by microfluidic platforms can form various microspheres whose components can be adjusted according to functional requirements. The composition and structure are altered to generate polymeric, inorganic, and metallic microparticles in the shape of sphere, nonsphere, and Janus.

#### 5.1.1 Spherical Functional Microparticles

##### Polymeric Microparticles

**Figure 6:**
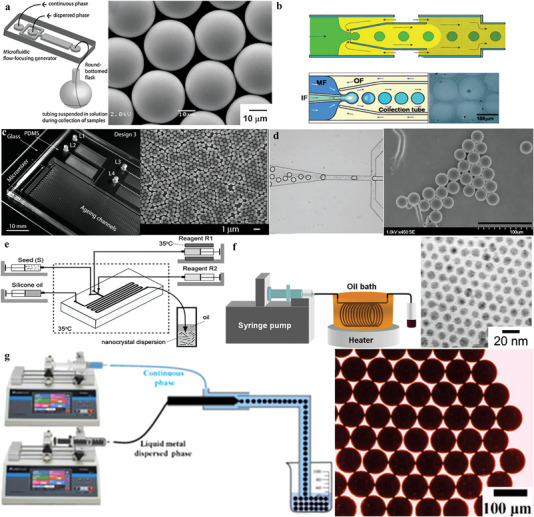
a) The preparation of PLGA monodisperse microparticles by flow focusing structure. Reproduced with permission.^[^ ^94^ ^]^ Copyright 2009, Wiley‐VCH GmbH & Co. KGaA, Weinheim. b) The preparation of uniform PNIPAM hydrogel microparticles based on glass capillary devices. Reproduced with permission.^[^ ^96^ ^]^ Copyright 2008, Royal Society of Chemistry. c) PDMS equipment for rapid synthesis of nanoscale colloidal silica. Reproduced with permission.^[^ ^107^ ^]^ Copyright 2004, American Chemical Society. d) The preparation of ordered mesoporous silica microparticles. Reproduced with permission.^[^ ^108^ ^]^ Copyright 2008, American Chemical Society. e) Ingenious preparation of gold nanoparticles suspended in an oily continuous phase using a PDMS‐based droplet microfluidic device. Reproduced with permission.^[^ ^118^ ^]^ Copyright 2009, Wiley‐VCH GmbH & Co. KGaA, Weinheim. f) Silver nanoparticles synthesized in a continuous‐flow tubular microfluidic device. Reproduced with permission.^[^ ^119^ ^]^ Copyright 2004, American Chemical Society. g) The generation of LM microparticles by flow focusing technique. Reproduced with permission.^[^ ^125^ ^]^ Copyright 2019, American Chemical Society.

Recently, microparticles composed of PVA,^[^ ^92^ ^]^ sodium alginate,^[^ ^93^ ^]^ PLGA,^[^ ^94^ ^]^ and polyethylene glycol (PEG)^[^ ^95^ ^]^ have been successfully used as drug carriers. By controlling the flow rates of two phases, PLGA monodisperse particles with good drug release capability in the range between 10 and 50 µm, which were generated by flow‐focusing geometry in a PDMS device (**Figure** **6**a). The Weitz research group^[^ ^96^, ^97^ ^]^ has selected the W/O single emulsion with a glass capillary‐based device (Figure 6b) to polymerize *N*‐isopropylacrylamide (NIPAM) monomer that dissolved in water droplets, and prepared PNIPAM hydrogel microparticles with uniform size. Similarly, Seo et al.^[^ ^98^ ^]^ have used O/W emulsion with a PDMS‐based device to prepare monodisperse polymer microparticles of different components and then triggered the polymerization of oil‐soluble monomers by ultraviolet light. When adding the controllable raw materials (such as dyes,^[^ ^99^ ^]^ magnetic nanoparticles^[^ ^100^ ^]^ or liquid crystals^[^ ^101^ ^]^) to organic materials, various functional properties can be imparted to microparticles. In addition to the effectiveness of microparticles for carrying chemotherapeutic or radio‐therapeutic agents to enhance therapeutic efficacy,^[^ ^102^ ^]^ magnetically guided drug carriers for therapeutic applications and medical imaging, which have been researched for decades, are also ready for clinical trials.^[^ ^103^ ^]^

##### Inorganic Microparticles

The monodisperse inorganic microspheres synthesized by droplet microfluidic technology contain ceramic particles such as silica and titania and have received considerable attention in the fields of catalysts,^[^ ^104^ ^]^ drug delivery,^[^ ^105^ ^]^ and microdevices such as sensors.^[^ ^106^ ^]^ Khan et al.^[^ ^107^ ^]^ have synthesized nanoscale colloidal silica (Figure 6c) by gas–liquid segmental flow in microchannels at a faster rate; furthermore, Carroll et al.^[^ ^108^ ^]^ proposed the microfluidic methods to fabricate the uniform ordered mesoporous silica microparticles with well‐defined (Figure 6d). Combining the diffusion‐induced self‐assembly technique, silica droplets constructed at flow‐focusing orifices with microparticles were generated by solidification around surfactant structures,^[^ ^109^ ^]^ and its corrugated surface morphology has the disordered mesoporous with an unprecedented size larger than 15 nm. Coincidentally, Wacker et al.^[^ ^110^ ^]^ used droplet microfluidics to graft submicron titanium dioxide particles with gold nanoparticles, which can greatly reduce the number of reagents and easily control the reaction. Under the droplets regime, monodisperse alginate microbeads^[^ ^111^ ^]^ with a narrow size distribution ≈60–95 µm (less than 3% of CV) have been produced in a droplet microfluidic device, which can be manipulated by varying flow rate, viscosity, and interfacial tension.

##### Metal Microparticles

Due to the size and shape dependent properties,^[^ ^112^ ^]^ metal microparticles play an extremely important role in metallic materials. As a result of a high ratio of surface area to volume, metal particles, especially in nanoscale, can be selected as effective catalysts for certain reactions. For noble metal particles with more stable chemical properties, they can be effectively used as good conductors in nanoelectronic devices. In addition, the special optical properties of noble metal nanoparticles enable to change their size and morphology for wave absorbing. Therefore, metal functional particles have the potential to be applied widely in catalysts,^[^ ^113^ ^]^ fine devices,^[^ ^114^ ^]^ optical materials,^[^ ^115^ ^]^ etc. Because the individual metal nanoparticle tends to aggregate and precipitate to reduce their surface free energy, traditional synthesis methods are hard to obtain microparticles in the desired size distribution range and the quality of mass production is unstable. Thermal and mass controls dictate the formation of metal particles, where droplet microfluidic devices offer a unique platform with fast heat and mass transfer rates.

Due to the good biocompatibility of gold nanoparticles, they have attracted the considerable attention in the field of application, and the demand has increased dramatically.^[^ ^116^ ^]^ Wagner and Köhler^[^ ^117^ ^]^ have developed a sandwich (glass–silicon–glass) structure for the synthesis of gold nanoparticles with 5–50 µm. In order to avoid the deposition of the generated particles on the inner wall, Duraiswamy and Khan^[^ ^118^ ^]^ ingeniously designed a PDMS‐based droplet microfluidic device to suspend the prepared particles in the oily continuous phase (Figure 6e). In the synthesis, the advantages of microfluidics are fully utilized, and the material consumption can be reduced by 95%. Lin et al.^[^ ^119^ ^]^ synthesized silver nanoparticles by using silver pentafluoropropionate as a single‐phase precursor in a continuous‐flow tubular microfluidic device. It was necessary to maintain the fabrication equipment at 100 °C, and the stainless‐steel hollow coil was immersed in an oil bath during the preparation process (Figure 6f). Further, Sotowa et al.^[^ ^120^ ^]^ improved the intersecting glass substrate channel into a triangular extension, and the silver nanoparticles were produced in the droplet formed by the collision of the two reactants. After modification, the tip of the stainless‐steel tube was inserted into the main microchannel directly rather than the wall, to prevent adhesion between wall and reaction reagents. Combined with the polyol process, Pt nanoparticles can be continuously prepared by a microfluidic device,^[^ ^121^ ^]^ and the particle size was influenced by pH of the synthesis solution. Moreover, the flow focusing technology can produce liquid metal (LM) particles with a diameter distribution in the range of 50–200 µm,^[^ ^113^ ^]^ shown in Figure 6g. The continuous phase can be aqueous (e.g., a mixture of glycerol and water or polyethylene glycol–electrolyte solution)^[^ ^122^, ^123^ ^]^ or oily (e.g., silicone oil or mineral oil),^[^ ^124^, ^125^ ^]^ and the addition of surfactants (e.g., polyvinyl alcohol)^[^ ^126^ ^]^ prevents them from agglomerating.

#### 5.1.2 Nonspherical Functional Microparticles

In addition to depending on its chemical compositions, the particle shapes also have a great influence on the function and application prospects of microparticles.^[^ ^127^ ^]^ With comparison to spherical microparticles, nonspherical ones have unique new functions; for instance, in terms of packing and packing, nonspherical microparticles can exhibit higher packing density than spherical microparticles.^[^ ^128^ ^]^ Therefore, it has played a very important role in drug delivery,^[^ ^129^ ^]^ tissue engineering,^[^ ^130^ ^]^ adsorption separation,^[^ ^131^ ^]^ etc. As the influence of interfacial tension remains the droplet as spherical as possible to keep system in a state of low interfacial energy, it is usually difficult to obtain nonspherical particles with uniform size by traditional batch polymerization methods. The precise manipulation by droplet microfluidic technology offers an excellent platform to control the preparation of nonspherical particles.

**Figure 7:**
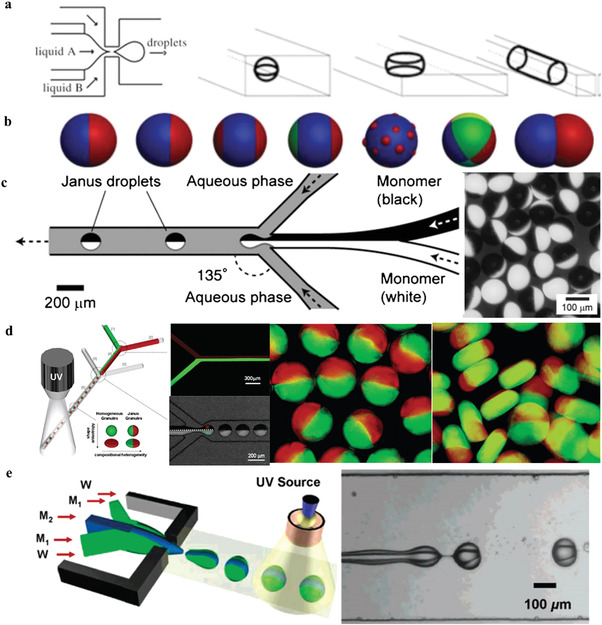
a. Fluidic microchannels of disk‐shaped, ellipsoidal or rod‐shaped nonspherical particles. Reproduced with permission.^[^ ^135^ ^]^ Copyright 2005, Wiley‐VCH GmbH & Co. KGaA, Weinheim. b) Janus particles in various shapes and structures. c) The preparation of Janus microparticles from acrylic monomers containing carbon black and titanium dioxide (TiO_2_) through Y‐shaped PDMS microchannels. Reproduced with permission.^[^ ^147^ ^]^ Copyright 2012, Royal Society of Chemistry. d) Microgel microparticles supported photocuring silicon particles. Reproduced with permission.^[^ ^140^ ^]^ Copyright 2006, American Chemical Society. e) The preparation of ternary microparticles under the jetting regime. Reproduced with permission.^[^ ^141^ ^]^ Copyright 2006, American Chemical Society.

One way is to confine droplets in the microchannels of varying sizes and shapes. If the droplet volume is larger than that of the maximum globe that can be accommodated in microchannel, it can deform into disk, rod‐shaped or ellipsoid nonspherical particles in the closed channel.^[^ ^132^, ^133^, ^134^ ^]^ Xu et al.^[^ ^135^ ^]^ designed the microchannel structure (**Figure** **7**a) so that the droplet with monomer solution flowing into microchannel was deformed into nonspherical shapes at the confined space. After curing in situ by UV light polymerization, the nonspherical polymer microparticles with rod and flat shape have been prepared in uniform size. In this approach, the certain volume of droplets contributes to the nonspherical microparticles with nearly uniform size and shape after aggregation. Additionally, during the polymerization process, the volume of material shrinks to a certain extent when it changes from liquid to solid, and the surface of the obtained microparticles still has an infiltrating liquid layer composed of a continuous phase liquid to isolate it from the microchannel, so it is effectively avoiding the blockage of microchannel by solid microparticles. Another way is to combine photochemistry and photomask with the pattern, and then photoinitiated polymerization occurs when droplets are periodically flowed through the mask. A variety of complex shapes have been easily produced, such as polygons, asymmetrical or curved objects, objects with high aspect ratios^[^ ^136^ ^]^ and nonspherical magnetic hydrogel microparticles.^[^ ^137^ ^]^ Moreover, the shape in the microchannel and various modes of UV light enable the fabrication of 4D complex structured particles that transform shapes with time changing. The mask only determines the shape and size of microparticles, while the chemistry and morphology can be independently selected for applications such as encoding, drug delivery, and biosensors. It displays the diversified characteristics of droplet microfluidic in the controllable preparation for nonspherical functional materials.

#### 5.1.3 Janus‐Shaped Functional Microparticles

Janus‐shaped functional microparticles consist of two parts, which can be completely different materials, or the same material with various functional groups on the surface or loaded with different organic/inorganic additives. In 1991, Nobel Prize winner Pierre‐Gille de Gennes introduced the concept of Janus particles to the scientific community.^[^ ^138^ ^]^ Asymmetric structures result in distinct physical or chemical properties (such as different surface wettability, magnetism, optoelectronic properties, etc.), so that individual particles are endowed with directionality. In terms of particle morphology, Janus microparticles can be grouped into various shapes and structures,^[^ ^99^ ^]^ such as multicompartment structures, as shown in Figure 7b. Droplet microfluidic technology can precisely control fluids operation under the laminar flow conditions, regarding as an excellent technical platform that is convenient and easy to scale up for the preparation of Janus‐shaped microparticles. In the fabrication process of droplet microfluidics, two types of liquid fluids with the same flowing direction are cut into the droplets, which can still maintain layer flow inside the droplets in a short period of time without mixing due to the dissolving.

In terms of flow focusing approach, the Y‐type PDMS microchannel is the most typical chip configuration to prepare Janus microparticles. Nisisako et al.^[^ ^139^ ^]^ took the lead in injecting the acrylic monomer with carbon black and titanium dioxide (TiO_2_) into two wings of Y channel in the upstream, respectively, which form a stable two‐phase stream in the main channel parallelly (Figure 7c). At the downstream cross‐section, the two‐phase parallel flow was sheared into Janus microdroplets by the continuous phase, where the relative flow velocity of the two fluids is an important factor in controlling the shape of the droplet. When the flow velocities are close, there is a relatively clear boundary between the two parts of the liquid droplet, while the convection occurring between two fluids will cause a mix for the large rate. After the subsequent offline thermalization is performed on the droplets, the single dispersion double colorless microparticles with a size ≈100 µm can be achieved. Owing to the hemisphere conductivity, they can be used in the color display field controlled by electric field. By following, Shepherd et al.^[^ ^140^ ^]^ prepared microgel loaded with silicon particles in the same way, whose two‐phase parallel flow contains two different silica particles of acrylamide aquatic solution. After broken into double‐sided liquid droplets, the acrylamide is rapidly aggregated under the UV light for polyacrylamide the Janus gel with the shape of sphere or disk (Figure 7d). In addition, Nie et al.^[^ ^141^ ^]^ obtained ternary microparticles under the jetting regime (Figure 7e). Similar to nonspherical microparticles, the size and shape of Janus ones can be controlled by the way of mask graphics to prepare nonspherical Janus structure in further. The research group of Doyle^[^ ^136^, ^142^, ^143^ ^]^ has proposed and studied thoroughly the functional nonregular Janus particles using microcontroller optical technology to prepare a variety of appearances. The application of nonsoluble fluids also allows the manufacture of Janus microparticles with half hydrophilic and half hydroponics, and the two ends form a sharp contrast in performance, involving the optical refractive index and bloating properties in different solvents. These microparticles enable to be used to transport and release hydrophilicity and organic macromolecule simultaneously.

Due to the demand for 4D properties of functional microparticles, Janus structures that can respond to a variety of different stimuli simultaneously have been prepared. Kim et al.^[^ ^144^ ^]^ have utilized the microfluidic chip and phase separation to fabricate Janus microspheres, in which the iron oxide nanoparticles (larger than 8 nm Hematite; *α*‐Fe_2_O_3_) in the photocurable resin ethoxylated trimethylolpropane triacrylate (ETPTA) is weakly ferromagnetic at room temperature, allowing nanoparticles to migrate within droplets via magnetophoresis. Notably, silica arrays on microparticle surfaces have the additional merit of having functionalities that can be chemically incorporated into chemicals or biomolecules. So, there is great potential in high‐throughput immunoassays and biological probes. Recently, Cui et al.^[^ ^145^ ^]^ synthesized magneto‐thermochromic coupled Janus microspheres for dual‐response display by focusing coflow technique, in which Fe_3_O_4_ microspheres and thermochromic particles were encapsulated by the polyacrylamide matrix. The Fe_3_O_4_ microspheres aggregated at one end can be rotated by controlling the external magnetic field, and when the ambient temperature increases from 5 to 45 °C, the thermochromic particles at the other end of microparticles can transfer their color from red to light blue due to the temperature dependence of the thermochromic particles. Furthermore, Wang et al.^[^ ^146^ ^]^ demonstrated the controllable droplet microfluidic way to assemble the Janus microparticles with temperature–magnetism–optics triple response, which was made up of polystyrene (PS) colloidal photonic crystals loaded with Cd^2+^ (photoresponse), *N*‐isopropylacrylamide (temperature response), and Fe_3_O_4_ nanoparticles (magnetic field response). By displaying different colors in response to various external fields, this technology could open the way for the design of more complex with multifunctional units and provide more potential applications in the environmental monitors, smart displays and anticounterfeit image codes.

### 5.2 Double and Multiple Emulsions

Multiple emulsions refer to the multiphase flow system formed by nesting smaller droplets inside the dispersed droplets.^[^ ^148^ ^]^ Compared with the single emulsion, these systems possess more types, which can encapsulate the gas inside.^[^ ^149^ ^]^ All the types of double emulsions have been shown schematically in Figure 5b, the most common of which are water‐in‐oil‐in‐water (W/O/W) and oil‐in‐water‐in‐oil (O/W/O) systems. The principle of double emulsion preparation by droplet microfluidics is to flow though the three phase into microfluidic device in an orderly manner, so that the inner phase is sheared by intermediate phase to form inner cores while generated droplets are sheared by the outmost continuous phase again. In addition, with the help of droplet microfluidics to control morphology and structure, the quantity of inner phase as well as size and thickness of outer phase can be regulated accurately. The microparticles with a high encapsulation rate ≈100%^[^ ^150^ ^]^ for the inner phase enable to provide a protected internal space for the encapsulation of substances, so it is widely applied in the field of drug delivery and release,^[^ ^151^ ^]^ synthesis of biomacromolecules,^[^ ^152^ ^]^ chemical catalysis,^[^ ^153^ ^]^ and others. According to the shape of the inner and outer shells, they are classified into hollow, core–shell, pore–shell, and multichambered functional microparticles.

#### 5.2.1 Core–Shell Functional Microparticles

The core–shell structure with the excellent chemical and physical properties is usually composed of an inner core and an outer shell, which are combined through the force between molecules. Based on microfluidic devices, the composition and particle size of core–shell particles can be designed in a targeted manner, as well as the cores of different components and sizes can be embedded, so that they have different characteristics such as magnetic, optical, and biological reactions. They have broad prospects in many different fields such as microreactors and tracer particles^[^ ^154^ ^]^ and so on. Generally speaking, the core and shell are composed of different materials; and while protecting the core from the external environment, the shell also has many other functions: 1) increase the mechanical strength and chemical properties of microparticles, 2) limit the volume change of individual microspheres to ensure integrity, 3) protect the core from agglomerating into large particles to ensure dispersion, and 4) restrict the selective entry of external ions into the core and protect the active core. The physical parameters of the fluid phase in the microchannel and the wall material are the key to the formation of the core–shell type. Considering multiple parameters such as the physical properties of the discrete phase, the wall effect, and the contact angle, Wang et al.^[^ ^155^ ^]^ carried out simulation calculations on the stability of droplets for the mechanism of core–shell formation under the shear action of continuous phase. They clarified the correlation among the stability of droplet formation, fluid parameters, and microchannel materials. In a broad sense, both 2D and 3D microfluidic devices for droplet‐based microfluidics can be used to construct the double‐emulsion droplets in a one‐step or two‐step method.

##### One‐Step Approach

**Figure 8:**
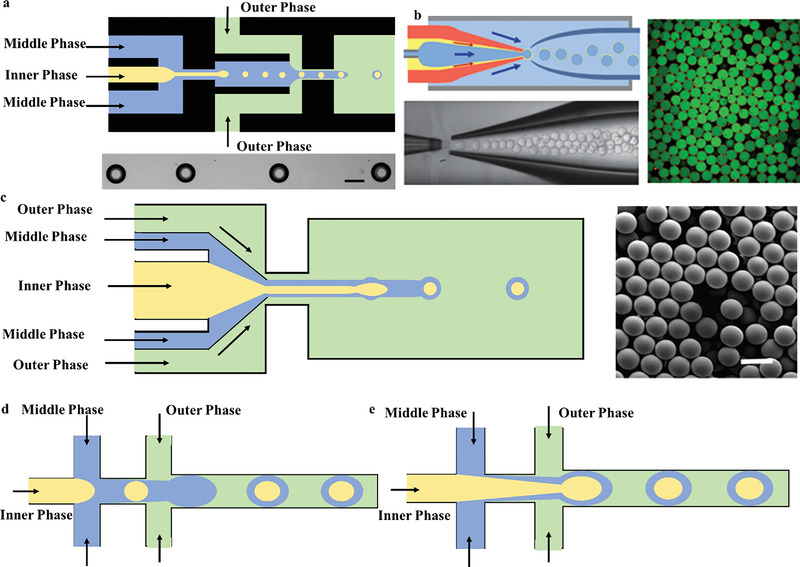
a) One‐step assembly of dual‐fluid focusing components to fabricate core–shell droplets. b) One‐step generation of double emulsion droplets with an ultrathin intermediate layer. Reproduced with permission.^[^ ^157^ ^]^ Copyright 2011, Royal Society of Chemistry. c) Two sequential flow‐focused droplet producers for generating core–shell droplets. Reproduced with permission.^[^ ^98^ ^]^ Copyright 2005, American Chemical Society. d) Two cross junctions that can achieve one‐step and two‐step formation.

*2D Device*: Nie et al.^[^ ^62^ ^]^ generated the core–shell droplets in one‐step assembly with double flow focusing parts on polyurethane by soft lithography (shown as **Figure** **8**a). In detail, the outer phase continuous fluid was introduced from two side microchannels, while the inner and middle phases were supplied from the middle microchannel. Due to the instability, the composite jet consisting of the inner and intermediate fluids is focused on the orifice, forming droplets in the downstream chamber. With the same equipment, Zhang et al.^156^ ^]^ obtained alginate capsules encapsulating cells, proteins, and enzymes with sizes ranging from 30 to 200 µm and CV below 4.0%.

*3D Device*: Utada et al.^[^ ^70^ ^]^ sequentially synthesized double‐emulsion droplets in a single step with coaxial focus (Figure 4g) using a custom‐fabricated glass capillary setup. It consisted of two cylindrical glass capillaries nested within a square glass tube; and the inner phase fluid was pumped through a pointed cylindrical tube, while the middle and outer phase fluids were pumped from either side of the square glass tube. The middle phase was coaxial with the inner one, and the outer phase was in the opposite direction to force all the fluids through the exit orifice formed by the remaining inner tube, breaking up to form droplets. To fabricate double emulsion droplets with an ultrathin intermediate layer in single step, Kim et al.^[^ ^157^ ^]^ created a feasible coaxial capillary microfluidic device (Figure 8b). One capillary was inserted into a round glass capillary with a tapered tip treated by a puller and they are secured in another square tube. Biodegradable polylactic acid microcapsules with a shell thickness in tens of nanometers were constructed at the outlet of the infusion tube for encapsulation and delivery of drugs, cosmetics, and nutrients.

##### Two‐Step Approach

In the two‐step method, the key factors are the formation of opposite wettability cascades and droplet formation geometries. The inner droplet is formed in the first droplet generator and then surrounded by the mesophase layer in the second droplet generator. Next, the core–shell microparticles can be solidified with freezing,^[^ ^158^ ^]^ solvent evaporation,^[^ ^159^ ^]^ ionic cross‐linking^[^ ^160^ ^]^ photo or thermal‐induced free‐radical polymerization^[^ ^161^ ^]^ can lead to core–shell microcapsules.

*2D Device*: By using two T‐junctions with a hydrophilic upstream and a hydrophobic downstream, Okushima et al.^[^ ^162^ ^]^ synthesized encapsulated droplets with O/W/O double emulsion or the first time in a glass microfluidic device (Figure 4b), whose CV is less than 4%. Seo et al.^[^ ^98^ ^]^ explored a PDMS microdevice with locally modified surface wettability, consisting of two sequential flow‐focused droplet producers (Figure 8c). W/O/W, O/O/W, and O/W/O double emulsions with precisely controlled droplet size were generated. The common setup was applied to form microcapsules containing an aqueous solution of aspirin in the core and an aqueous solution of high‐molecular‐weight chitosan loaded with Fe_3_O_4_ nanoparticles in the shell,^[^ ^154^ ^]^ which were cross‐linked with glutaraldehyde to achieve a solid shell with magnetic response.

*3D Device*: As shown in Figure 4d, this two‐stage coaxial microfluidic device was composed by three round tubes and two square tubes. The internal phase is sheared by medial phase to arrange dispersed and independent microdroplets that enters the second‐stage pipeline, wrapping by the faster‐flowing external phase liquid to form microspheres. Usually, the middle phase is based on UV‐polymerizable monomers (such as ethylene dimethacrylate^[^ ^163^ ^]^), surfactants and photoinitiators, etc., so that the droplets can be cured under UV light irradiation.

The device composed by two cross junctions^[^ ^164^ ^]^ can achieve one‐step and two‐step formation by controlling the flow rate (Figure 8d,e). Taking the W/O/W emulsion as an example, the inner aqueous droplets are generated at the first intersection and are encapsulated in the intermediate oily droplets at the second intersection. The coaxial jets of the inner and middle phases extend into the outer phase where it breaks up for core–shell microparticles. It is easier to perform one‐step double emulsion formations since precise patterning is not as necessary as in two‐step methods. Once forming the internal jet, it is surrounded by the intermediate fluid, favoring wetting even at the first junction. Abate et al.^[^ ^165^ ^]^ verified the feasibility of the design with flow confinement to simulate the wettability of PDMS, involving inert fluid, thermal initiation, and photoinitiation. Similar to the formation of double emulsions, multiple emulsion microparticles can be generated by multiple‐ or one‐step emulsification method. Utilizing the core–shell structure is aimed at performing directed differentiation of microcarriers that was composed by stem cells as the core and differentiated cells as the surrounding layer, respectively. As the shell contains functional beads or drugs, it has the potential for fluorescent labeling, drug delivery or magnetic manipulation.^[^ ^166^ ^]^

#### 5.2.2 Pore–Shell Functional Microparticles

**Figure 9:**
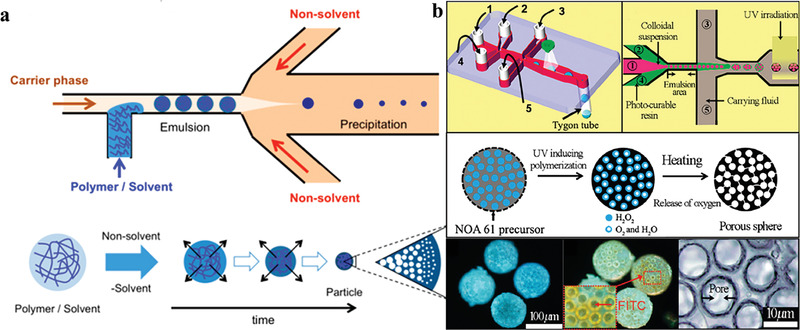
a) Microfluidic emulsification followed by solidification of solvent‐extracted droplets to produce internally porous polymer microparticles. Reproduced with permission.^[^ ^169^ ^]^ Copyright 2014, American Chemical Society. b) Microfluidics of microporous polymer microparticles with precursor solidification and oxygen release simultaneously. Reproduced with permission.^[^ ^171^ ^]^ Copyright 2019, MDPI.

For the efficient mass transfer process between microparticles, material molecules can be promoted to pass through the shell by constructing a pore structure on the microparticle shell. The encapsulation and controlled release process of substances are further controlled by the size and functionality of the pores,^[^ ^167^ ^]^ so that the functions of microparticles are more diversified. Due to low density, large specific surface area, great porosity, and strong permeability, porous structure microspheres are widely used in adsorption, especially in the fields of catalysis, energy storage. Regardless of the template method or the addition of pore‐forming agents, the pore size deviation of the microspheres prepared by the microfluidic device is small, while the pore size is the significant factor affecting selective adsorption of the microparticles. As the porosity increases, more adsorption sites are released not only on the surface of microparticles, but also inside the spheres, thereby building up the adsorption capacity of microspheres. Wang et al.^[^ ^168^ ^]^ prepared chitosan porous microspheres with uniform morphology by droplet microfluidics method, and their adsorption capacity of heavy metal ions increased by three times as a result of adding polyethyleneimine for more adsorption sites to enhance chemical adsorption capacity. As shown in **Figure** **9**a, Watanabe et al.^[^ ^169^ ^]^ introduced a method using microfluidic emulsification followed by solidification of solvent‐extracted droplets to produce internally porous polymer microparticles. In this process, a thiolene droplet microfluidic device was employed instead of PDMS, which improved the resistance to organic solvents and enabled the use of common organic solvents (including hexadecane) as the continuous phase^[^ ^170^ ^]^ Meanwhile, it was demonstrated that the initial polymer concentration and droplet viscosity determine the particle porosity. Using a flow focusing microfluidic device, Amoyav and Benny^[^ ^171^ ^]^ synthesized porous PLGA and poly(d,l‐lactide) porous microparticles with higher precision and narrower size distribution. Resemble to the fabrication of core–shell microparticles, the high introversion composite emulsion system can be applied as templates by using the volatilization of gases, such as H_2_O_2_, NH_4_HCO_3_, etc. The solution is mixed in the inner phase, and will be decomposed into gases insoluble in microspheres under light conditions, such as: O_2_, CO_2_, NH_3_, N_2_, etc. Amoyav and Benny^[^ ^171^ ^]^ introduced the microfluidic fabrication of macroporous polymeric microspheres by simultaneous UV‐induced polymerization of the shell and decomposition of H_2_O_2_, and the precursor solidifies while releasing oxygen, forming a porous structure (Figure 9b). The controllable flow rate can not only adjust the particle size, but also design the number and size of pores inside the polymer.

#### 5.2.3 Controllable Chamber Functional Microparticles

##### Single‐Chamber Functional Microparticles

Functional microparticles with a single‐chamber structure that possesses the function of substance encapsulation and releasing are widely applied in the field of encapsulating and protecting substance, drug analysis and release, miniature reaction, etc. Generally, single‐chamber microparticles can be synthesized through the processes of polymer self‐assembly, phase separation, and layer‐by‐layer assembly based on droplet or particle templates. In order to achieve a hollow structure, the following methods can be used.

- Water soluble reaction

Zhang et al.^[^ ^172^ ^]^ proposed an O/W/O double emulsion method with glass capillary device to mix NIPAM (temperature‐sensitive properties), 3‐acrylamidophenylboronic acid (glucose recognition properties), and hydrophilic acrylic monomer into an aqueous solution of the middle phase, and then initiating polymerization by ultraviolet light. After the internal oil droplets were washed away with an organic solvent, monodisperse glucose‐responsive hydrogel microparticles with a hollow cavity structure were prepared. It provides a new model and theoretical guidance for the design and development of new self‐regulated controlled‐release carriers for diabetes treatment. Based on this droplet microfluidic preparation method, the researchers flexibly adjusted the functional materials in the aqueous solvent of middle phase, such as *N*,*N*‐dimethylaminoethyl methacrylate, NIPAM or 4‐Acryloylamidobenzo‐18‐crown‐6. Also, single‐chamber hydrogel microparticles that can respond to pH changes^[^ ^173^ ^]^ or lead ion concentration changes^[^ ^174^ ^]^ to achieve shell swelling and contraction have also been successfully prepared, with a view to control the release of substances under different demands.

- Interfacial cross‐linking

**Figure 10:**
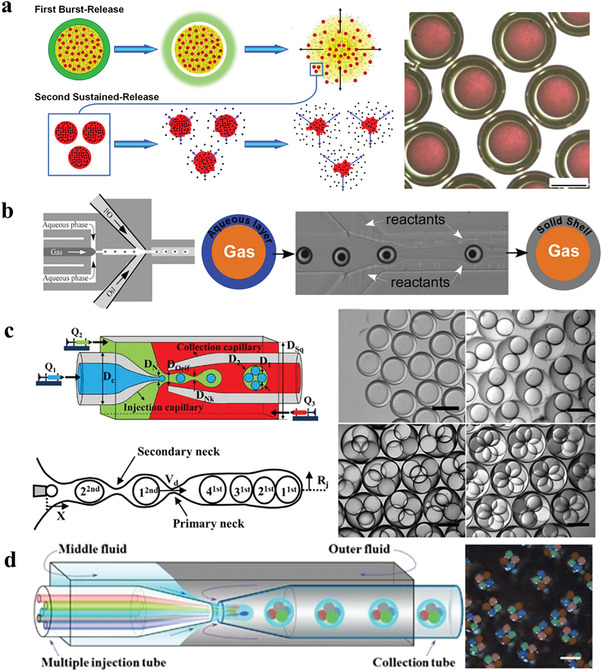
a) Construction of single‐compartment chitosan microparticles with sequential drug release function via interfacial cross‐linking reaction. Reproduced with permission.^[^ ^175^ ^]^ Copyright 2016, American Chemical Society. b) Synthesis of inorganic hollow particles by double emulsion with coflowing method. Reproduced with permission.^[^ ^177^ ^]^ Copyright 2011, American Chemical Society. c) Single‐step generation of monodisperse multinuclear double emulsion droplets in a three‐phase glass capillary microfluidic device. Reproduced with permission.^[^ ^179^ ^]^ Copyright 2017, Elsevier B.V. d) The preparation of multiple microparticles in a droplet microfluidic device equipped with multiple internal phase microchannels. Reproduced with permission.^[^ ^181^ ^]^ Copyright 2012, Spring Nature.

Microparticles with a single‐chamber structure can be constructed by solidifying the outer layer droplets containing functional materials in the double emulsion into capsule walls. With microfluidic template, Yang et al.^[^ ^175^ ^]^ took advantage of the O/W/O double emulsion to construct single‐chamber chitosan microparticles with sequential drug release function through interfacial cross‐linking reaction. It was composed by an outer shell of chitosan hydrogel and an inner oil core that contains free drug molecules and drug‐loaded PLGA nanoparticles. The chitosan shell can be dissolved under acidic conditions, thereby rapidly releasing the internal substances in a short time (≈60 s), as shown in **Figure** **10**a. These chitosan microparticles with hollow structure can not only realize the synergistic encapsulation of different drugs, but also realize the coupling of different release modes, and the acid‐triggered sequential drug release function has a good application prospect in the treatment of acute gastritis. In addition to the method similar to the above, Yang at al.^[^ ^176^ ^]^ also added Fe_3_O_4_ nanoparticles to the middle phase and utilized the interfacial cross‐linking reaction to gel the chitosan molecules in the water phase. Thus, the hollow structure chitosan microparticles with multiple stimuli‐responsive drug‐controlled release functions were controllably constructed.

- Wrapping gas directly

Wan and Stone^[^ ^177^ ^]^ proposed the coflowing approach with G/W/O double emulsions to synthesize inorganic hollow particles, in which the middle phase is deionized water mixed with glycerol, and the outer phase is a low concentration of PDMS oily solvent (Figure 10b). Single‐chamber particles with silica or titania are fabricated in one continuous flow process by introducing reactive inorganic precursors (such as silanes and metal alkoxides) to perform sol–gel reactions downstream of bubble formation in the droplets. Microemulsions generate a limited amount of water in a controlled manner, which avoid the unpredicted hydrolysis reactions in the sol–gel process. In particular, the increased viscosity of the aqueous phase contributes to encapsulate the individual air bubbles in the water droplets with the ability to control shell thickness.

##### Multiple Chamber Functional Microparticles

Multichamber microparticles can separate and encapsulate substances of different components and can realize on‐demand release of encapsulated substances. As a delivery vehicle, it is of great importance for the coordinated transport of incompatible active substances. Droplet microfluidic multiple emulsions provide a unique template for the design and preparation of multichamber functional microparticles. Till present, Chu et al.^[^ ^82^ ^]^ used the separation of two emulsification steps to provide independent control of each step to control the quantity of droplets; as shown in the, the innermost layer and the middle layer can wrap up to 7 and 3 droplets, respectively. With the similar method, Huang et al.^[^ ^178^ ^]^ generated polynuclear alginate gel capsules for encapsulating hydrophobic drugs to explore the release of *α*‐tocopherol. In addition, Nabavi et al.^[^ ^179^ ^]^ studied the one‐step generation of multinuclear microparticles through a three‐phase glass capillary microdevice (Figure 10c), and the maximum number of stable inner droplets that can be formed is six. For more versatile two‐component gas formers, Duncanson et al.^[^ ^180^ ^]^ proposed the application of a dual‐hole microdevice to isolate the reactants of different gas‐forming reactions until they were encapsulated in an outer droplet. The characteristic of droplet microfluidic encapsulation of droplets with different components also provides the possibility to integrate materials with different functions into the same microparticle to obtain multifunctional properties. Zhao et al.^[^ ^181^ ^]^ reported a class of barcodes prepared by a droplet microfluidic device equipped with multiple internal phase channels (Figure 10d), which includes multiple photonic crystals or magnetically labeled ETPTA core and PEG shell. Based on their periodic structure, these stable codes exhibit identical photonic and magnetic responses, promising as microcarriers in biomedical applications that involve high‐throughput bioassays and cell culture studies requiring multiplexed analysis.

## 6 Emerging Application

### 6.1 Additive Manufacturing

**Figure 11:**
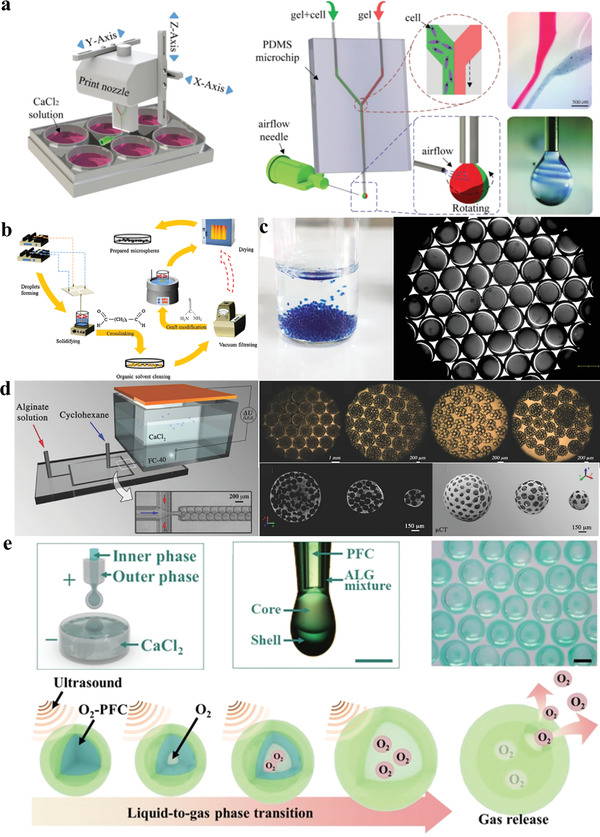
a) The combination of droplet microfluidics and additive manufacturing to prepare helical microparticles. Reproduced with permission.^[^ ^178^ ^]^ Copyright 2018, Wiley‐VCH GmbH & Co. KGaA, Weinheim. b) Generation of thiourea‐modified chitosan microspheres as biosorbents. Reproduced with permission.^[^ ^183^ ^]^ Copyright 2016, Elsevier B.V. c) The production of microcapsules for carbon capture by needle‐based microdevices. Reproduced with permission.^[^ ^184^ ^]^ Copyright 2019, Elsevier Ltd. d) Preparation of microbeads with porous structure by flow focusing microfluidic device. Reproduced with permission.^[^ ^199^ ^]^ Copyright 2018, Wiley‐VCH GmbH & Co. KGaA, Weinheim. e) Combining glass capillary microfluidic device with ionic cross‐linking to prepare microcapsules for codelivery of oxygen and drugs. Reproduced with permission.^[^ ^203^ ^]^ Copyright 2022, Elsevier Ltd.

The conditions of generation process can be far away from high voltage and high temperature, which can effectively protect the endowed function, especially for biological cells.^[^ ^182^ ^]^ These unique properties enable monodisperse microfluidic functional materials for various applications in medical additive manufacturing. Incorporating airflow assistance, Zhao et al.^[^ ^183^ ^]^ presented a novel bioprinting method for producing helical spheres with excellent resolution and complex microstructures, enabling novel biomimetic asymmetric prototypes for basic medical research and regenerative medicine. As shown in **Figure** **11**a, the PDMS microfluidic chip was selected as the primary part of the nozzle in the direct ink writing, and the Y‐shaped microchannel allows two or three parallel bioinks to be extruded from a single nozzle with high resolution and fast response. A dispensing needle was placed at the exit of nozzle exit that closed to the generating droplet. Under the action of airflow, the originally parallel sodium alginate solution loaded with cells/blanks in the droplets gradually formed a helical structure. After being dropped into a container filled with CaCl_2_ solution, a thin layer of calcium alginate quickly formed on the surface of the droplet, thereby maintaining the integrity of the internal structure.

### 6.2 Environmental Treatment

In recent years, environmental pollution has become a severe challenge for human beings, especially the environment and water resources that depend on for survival. Whether dealing with water pollution, air pollution, or soil pollution, adsorption has been a practical and inexpensive and well‐established technology.^[^ ^184^ ^]^ The conventional technologies might not degrade or degrade incompletely during the treatment process, and even the intermediate substances produced will cause secondary pollution. The removal of heavy metals with ion exchange or resin chelation leads to the concentration of a large number of toxic and harmful elements on the surface of adsorbed material.^[^ ^185^ ^]^ Adsorbents with surface charges can cause harm to humans in addition to multiple detrimental effects on the environment.^[^ ^186^ ^]^ Since droplet microfluidics can effectively control the spatial structure of the emulsion during the preparation process, the addition of foaming agent PEG increases the specific surface area of microspheres and improves the adsorption rate in sewage treatment.^[^ ^187^ ^]^ Zhu et al.^[^ ^188^ ^]^ synthesized a thiourea‐modified microspherical biosorbent with polyethylene glycol added to chitosan aqueous solution as the main component (as shown in Figure 11b). The copper‐ion adsorption experiments proved that it had a higher adsorption capacity (*q*_e_ = 60.6 mg g^−1^) with thiourea modification added amino functional groups on the surface of the microspheres, which was comparable to that of unmodified chitosan microspheres. Moreover, Lian et al.^[^ ^75^ ^]^ prepared porous PDMS particles through a needle‐based microfluidic device with O/W single emulsion mode, in which tetrachloromethane (CCl4) and white granulated sugar were selected as porogens, respectively. The manufactured microparticles have been applied to treat wastewater containing toluene in the static and dynamic processes, and more than 65% of the pollutants have been removed. Through the regeneration test, the microparticles are competent after about 30 times of recovery efficiency. To effectively reduce carbon, Yew et al.^[^ ^74^ ^]^ first took advantage of an off‐the‐shelf needle‐based microdevice (Figure 3) with W/O/W emulsion to produce microcapsules for carbon capture, typically ranging of 600–720 µm and a CV of 0.97–3.0% (as shown in Figure 11c).

### 6.3 Food Industry

The food and nutrition sector faces major challenges related to the quantity, safety, and quality of a global population of more than 7 billion people. To this end, food design needs to consider from the role of food composition and structure to food physicochemical stability and food digestion, optimizing nutrient delivery in the body. The processes that determine the structure and behavior of food during production, storage, and final digestion occur at the nano‐ and microscales. The detection of food safety by microfluidic devices is mainly achieved through the quantification of pathogen cells or pathogen‐associated metabolites such as DNA. Currently, a microfluidic flow cell with embedded arrays of gold interdigitated microelectrodes, integrated with magnetic nanoparticle–antibody conjugates, is able to detect *Escherichia coli* in meat products in 35 min,^[^ ^189^ ^]^ while traditional methods take more than 24 h. To realize on‐site food safety detection, microfluidic devices have also been applied to detect other foodborne pathogens, such as Listeria,^[^ ^190^ ^]^ Norovirus,^[^ ^191^ ^]^ and Salmonella.^[^ ^192^ ^]^ In terms of food manufacturing, microcapsules protect food ingredients from heat, oxidation, maintaining a strong mean of moisture and pH. By encapsulating food ingredients, texture, aroma, and flavor release can be effectively controlled and less fatty and oily food can be produced.

### 6.4 Tissue Engineering

Tissue engineering is an interdisciplinary field aimed at regenerating diseased tissue, and it can be grouped into two categories with cells and scaffolds. To achieve predictable and highly efficient implants, integration of nano‐ and microtechnology into macroscopic natural biomaterials enables spatiotemporal control of the microenvironment. Given that the 3D culture system can provide a more precise biomimetic environment, the droplet microfluidic system can contribute to the 3D culture of stem cells.^[^ ^193^ ^]^ By mimicking the niche of stem cells for cell therapy purposes, in the aspect of neural tissue, enhanced neural migration and differentiation and monitoring have been achieved.^[^ ^194^ ^]^ Liu et al.^[^ ^195^ ^]^ utilized a droplet microfluidic system to fabricate hybrid hydrogel capsules under control, allowing large‐scale 3D culture of human induced pluripotent stem cells for pancreatic islet organoids. In addition, droplet microfluidics provides a controlled microenvironment for gamete, fertilization, and embryo culture in the field of reproductive medicine.^[^ ^196^ ^]^ Injecting scaffolds loaded with cells in the form of mesoscopic granules directly into the treatment site is a method that facilitates tissue regeneration.^[^ ^197^ ^]^ As a degradable biological structure, scaffolds can provide temporary support for cells while new tissue grows. Wang et al.^[^ ^198^ ^]^ developed a method of using a microfluidic device to produce highly organized single polymer (alginate) scaffolds for the treatment of osteoarthritis. Next, Costantini et al.^[^ ^199^ ^]^ proposed the preparation of 300–1500 mm microbeads (Figure 11d) with a uniform and fully interconnected internal porous structure by means of flow‐focusing microfluidic devices, from which porous bead scaffolds can be precisely produced. Improvements in the design and high‐throughput performance of droplet microfluidic devices have overcome the difficulty of combining millimeter‐scale models with appropriate vasculature and connecting them to effective microphysiological systems. Further research can realize its potential application in different experimental platforms of tissue engineering.

### 6.5 Biomedical Applications

Micro/nanomaterials have received the extensive attention in the field of biomedical engineering, such as diagnostics, pharmaceuticals, and therapeutics. Taking advantage of droplet microfluidics to precisely adjust the geometry and tunable properties of particles,^[^ ^200^ ^]^ it can provide effective materials for biomedical applications. During the early droplet microfluidic biosensors can be used to monitor biomarkers,^[^ ^201^ ^]^ including hormones, metabolites, bacteria, etc. In therapy, drug delivery systems (DDSs) have achieved targeted delivery to release drugs to designated locations.^[^ ^202^ ^]^ The ideal DDS material size requires customized and controllable properties, while droplet microfluidics can stably generate single or multiple emulsion droplets for controlled release of hydrophobic and hydrophilic drugs. Huang et al.^[^ ^203^ ^]^ combined glass capillary microfluidic devices and ion cross‐linking to prepare microcapsules for codelivery of oxygen and multidrugs (Figure 11e), and then improved chemo‐sonodynamic therapy through the original injection method. In addition, microfluidic technology can fabricate stimuli‐responsive biodevices with sensing functions, such as flexible electronics and soft robots, to monitor various physiological signals of human health in a wireless mode.^[^ ^204^ ^]^ Complex structural capabilities, encapsulation and efficient release, and high throughput with relevant productivity enable biomaterial applications. Under rational design, there are challenges and promises in fabricating materials with in‐demand properties and functions for biomedical applications.

## 7 Conclusions

This paper reviews the defects and bottlenecks of conventional methods in the preparation of functional microparticles, and highlights the convenience and advantages of droplet microfluidic technology. The preparation process can not only adjust size, shape, as well as shell thickness of microparticles, but also precisely control the structure, morphology, and components inside the microparticles. By summarizing the progress of droplet microfluidic devices in the past two decades, the revolutionary breakthrough from 2D to 3D has brought opportunities for multifunctional particles. Through different extrusion modes, single emulsion or multiple emulsion methods enable to fabricate spherical, nonspherical, and Janus microparticles, as well as core–shell, pore–core, and controllable chamber microparticles. The ingenious combination of microparticle structure and functional components constituting microparticles endows microparticles with more diversified functions, thus providing new ideas and guidance for the design and development of functional microparticles.

To date, despite amount of the exciting and compelling academic achievements have been got in the field of droplet microfluidics, there are still challenges to be addressed, which have potential for further development. At present, most functional microparticles are basically produced drop‐by‐drop, which cannot be mass‐produced with the limitation of productivity. In order to promote further innovation and industrial application in emerging fields, it is necessary to conduct in‐depth and systematic research on the combination of additive manufacturing and droplet microfluidics to achieve bioprinting diversity. It is also the main direction of future microfluidic technology research to investigate how to improve the size ratio between core and shell, and tune the structure of microparticles so that they have the ability to encapsulate a variety of active materials.

## Conflict of Interest

The authors declare no conflict of interest.
